# Groundwater Impacts from the M5.8 Earthquake in Korea as Determined by Integrated Monitoring Systems

**DOI:** 10.1111/gwat.12993

**Published:** 2020-03-18

**Authors:** Soo‐Hyoung Lee, Jae Min Lee, Heesung Yoon, Yongcheol Kim, Seho Hwang, Kyoochul Ha, Yongje Kim

**Affiliations:** ^1^ Korea Institute of Geoscience and Mineral Resources 124 Gwahak‐ro, Yuseong‐gu Daejeon 34132 Republic of Korea

## Abstract

This paper describes the impacts of the M5.8(5.1) Gyeongju earthquakes on groundwater levels using data obtained from a unique coastal monitoring well. The monitoring strategy integrates conventional water level monitoring with periodic, continuous measurements of temperature and electrical conductivity (EC) within the water column of the well. Another important component of the monitoring system is a new instrument, the InterfacEGG, which is capable of dynamically tracking the freshwater‐saltwater interface. Although the system was set up to monitor seawater intrusion related to over‐pumping, as well as rainfall and tidal effects, it recorded impacts associated with a large earthquake and aftershocks approximately 241 km away. Seismic energies associated with the M5.8(5.1) Gyeongju earthquakes induced groundwater flows to the monitoring well through fractures and joints in the crystalline basement rocks. Temperature and EC logging data showed that the EC vertical profile declined from an average of approximately 5300 to 4800 μS/cm following the earthquakes. The temperature profile showed a trend toward lower temperatures as the depth increased, a feature not commonly observed in previous studies. Data from the InterfacEGG suggested that the rise in EC was not due to the saltwater intrusion, but from the tendency for brackish water entering the borehole to induce convective mixing at deeper depths as the seismic waves travel through the well‐aquifer system. The increase in groundwater levels was caused by pulse of colder, less brackish water flowing into the well because of the earthquake. This behavior reflects an enhancement in rock permeability by removing precipitates and colloidal particles from clogged fractures, which improve the hydraulic connection with a nearby unit with a higher hydraulic head. This study suggests there is value added with a more aggressive monitoring strategy.

## Abstract 2

*Article impact statement*: This paper describes the impacts of the M5.8(5.1) earthquakes on groundwater using data obtained from a novel integrated monitoring system. The study illustrates the value added by using continuous well logging rather than single‐depth measurements of water level change. In addition, a new instrument, InterfacEGG, proved to be useful in monitoring the freshwater‐saltwater interface. This new and innovative approach provided convincing evidence of the impact of the main earthquake on the groundwater system. Although our application herein was specifically concerned with changes associated with an earthquake, it demonstrates the potential usefulness of such an integrated system for monitoring the dynamic behavior of coastal aquifers.

## Introduction

Groundwater wells installed in coastal aquifers commonly monitor changes in groundwater conditions related, for example, to rainfall, tidal effects, pumping, and even earthquakes (Ferris 1951). Earthquakes and seismic waves have been reported to induce changes in aquifer properties associated with fluid pressures (King et al. 1999; Chia et al. 2001; Roeloffs et al. 2003; Shi et al. 2007; Wang and Chia 2008), which can lead to significant changes in the hydrogeological characteristics of aquifers (Rojstaczer et al. 1995; Jonsson et al. 2003; Wang et al. 2009). King et al. (1981) found that crustal strain led to rock fracturing that in turn caused changes in salinity and EC of the groundwater by mixing with seawater. More specifically, seismic waves traveling through rock activate microcracks and microparticles within fracture zones. Changes can occur to physical and chemical properties, such as temperature and water chemistry by readjustments in groundwater flow, water‐rock interaction and fluid‐source mixing (Claesson et al. 2004, 2007; Wang et al. 2012; Skelton et al. 2014). In karst aquifers, Charmoille et al. (2005) found that once groundwater levels experienced seismic waves, mixing between old water and younger water with shorter residence times, produced changes in EC.

Seismic waves are also known to critically affect groundwater levels and water chemistry in aquifers, as determined by monitoring of groundwater wells located thousands of kilometers away (Cooper et al. 1965; Liu et al. 1989; Brodsky et al. 2003; Sil and Freymuller 2006; Wang et al. 2009; Lee et al. 2013). However, these previous studies have relied on measurements with single wells of fixed depths to determine changes in groundwater levels and water quality. Such results are limited because they, in essence, provide groundwater data at only a single observation point for an earthquake.

The purpose of this study is to demonstrate the efficacy of a new observational approach that is capable of providing a vertical profile of changes in temperature and EC as well as groundwater levels. The data here relating to the M5.8(5.1) Gyeongju earthquakes were collected at a monitoring well used principally to monitor saltwater intrusions. The results of this study shed new light on groundwater impacts of earthquakes in coastal aquifers. Moreover, it highlights the innovative features of this monitoring strategy, which is obviously beneficial to the management of coastal aquifers because it yields clearer understanding of freshwater and saltwater flows.

## Monitoring Well Design and Measurements

The study area, Seacheon, is located on the west coast of the Korean peninsula (Figure ^1^a), containing a thin veneer of unconsolidated sediments that overlie crystalline bedrock (Figure ^1^b). The monitoring wells are located at approximately 60 m inland from the shoreline with the elevation of the monitoring wells (MW1, MW2, and MW3) of 5 m above sea level (Kim et al. 2019), and borehole depths are 100, 150, and 30 m, respectively (Figure ^1^c and ^1^d).

**Figure 1:**
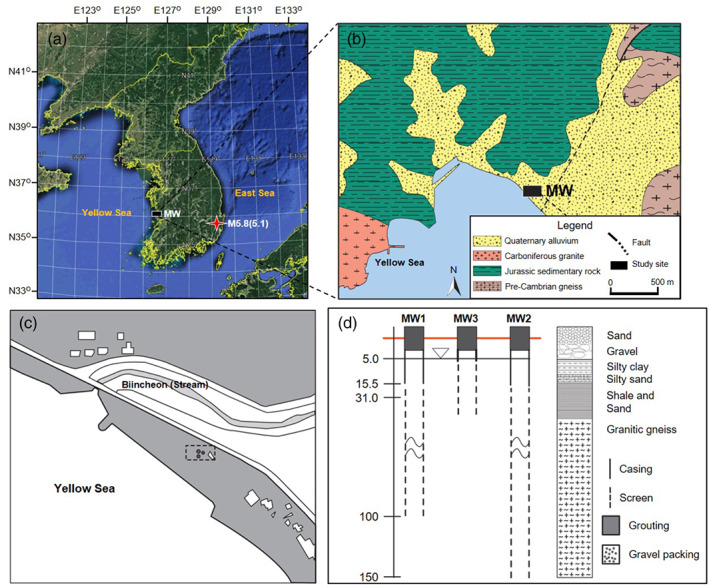
Location of the study area showing (a) locations of epicenter of the M5.8(5.1) Gyeongju earthquakes and monitoring wells (MW), (b) a geologic map of the area nearby the MWs, (c) an enlargement of the MWs area, and (d) the geological vertical section of the MWs.

Groundwater level and barometric pressure were measured at 1‐min intervals in one of the three monitoring wells (MW1). In addition, both temperature and EC were measured to examine the behavior of the saltwater‐freshwater interface through times. Essentially, vertical logging of temperature and EC provide the location of the freshwater‐saltwater interface level (FSL) and allows for the analysis of groundwater flows in coastal aquifers (Swanberg et al. 1988; Helm‐Clark et al. 2004).

The location of the FSL and the thickness of overlying fresh water were measured using a wireless pressure sensor, InterfacEGG, and a pressure sensor at a fixed depth (Figure ^2^). The InterfacEGG is a monitoring probe designed with a solid density that is greater than freshwater and less than saltwater. Thus, it floats within freshwater‐saltwater interface (Kim et al. 2016). In other words, by fixing the density appropriately in relation to the surrounding fluids, the probe has neutral buoyancy. It stays in place without sinking or rising if no external forces exist. Accordingly, the probe moves up and down, tracking the movement of the freshwater‐saltwater interface. Using measured pressure data from the InterfacEGG and a fixed pressure sensor at depth, the thickness of the freshwater and the time series of the FSL can be estimated as follows:

$$\text{Depth to Interface}\;d\left(t\right)=\left[b-a\left(t\right)\right]+c\left(t\right)$$

$$\text{Thickness of Freshwater}=c\left(t\right)$$

where *a*(*t*) is the pressure of the freshwater column above sensor A (variable), *b* is the depth to sensor A (fixed), *c*(*t*) is the pressure above the interface (InterfacEGG, variable), and *t* is time.

**Figure 2:**
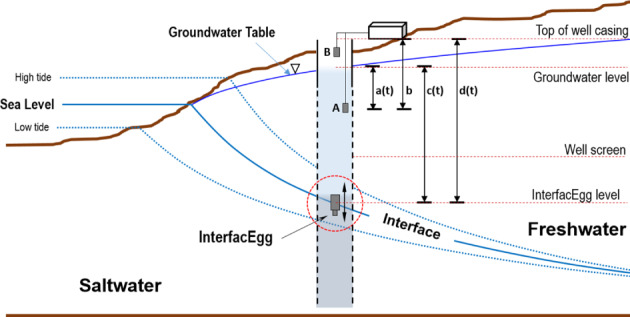
Schematic diagram showing the FSL depth, the monitoring system, and reference distances used to calculate the FSL depth.

## Results and Discussion

### Gyeongju Earthquakes and Aftershocks

Earthquakes of magnitude M5.1 and M5.8, as moment magnitudes, occurred in the vicinity of Gyeongju, a historic city located on the southeast coast of South Korea at 10:44:32 UTC and 11:32:54 UTC, respectively, on September 12, 2016. Following the M5.8 earthquake, there were 157 aftershocks with magnitudes >2.0 and 30 aftershocks >3.0 that continued until November 13, 2016. Table ^1^ provides the timing of foreshocks, the main events and largest aftershocks. The M5.8 earthquake is the largest recorded earthquake in Korea since the advent of modern recording systems. Shown in Figure ^1^a is the epicenter located approximately 241 km away from the seawater intrusion monitoring well to the west.

**Table 1:** Time‐Line of Events for Gyeongju Earthquakes Greater than a Magnitude M3.0

Earthquake		Magnitude (M)^1^	Distance (km)	Date (Local Time)	Date (UTC)
Gyeongju	Foreshock	5.1	240 to 242	2016.09.12 19:44:32	2016.09.12 10:44:32
		3.1		2016.09.12 19:48:03	2016.09.12 10:48:03
		3.1		2016.09.12 20:10:50	2016.09.12 11:10:50
	Main earthquake	5.8		2016.09.12 20:32:54	2016.09.12 11:32:54
	Aftershock	3.6		2016.09.12 20:34:22	2016.09.12 11:34:22
		3.4		2016.09.12 20:36:00	2016.09.12 11:36:00
		3.0		2016.09.12 20:38:00	2016.09.12 11:38:00
		3.0		2016.09.12 20:39:35	2016.09.12 11:39:35
		3.0		2016.09.12 20:40:40	2016.09.12 11:40:40
		3.0		2016.09.12 23:18:27	2016.09.12 14:18:27
		3.1		2016.09.12 23:52:30	2016.09.12 14:52:30
		3.1		2016.09.13 00:37:10	2016.09.12 15:37:10
		3.2		2016.09.13 08:24:47	2016.09.12 23:24:47
		3.0		2016.09.13 14:31:42	2016.09.13 05:31:42
		3.0		2016.09.14 00:48:41	2016.09.13 15:48:41
		4.5		2016.09.19 20:33:58	2016.09.19 11:33:58
		3.5		2016.09.21 11:53:54	2016.09.21 02:53:54
		3.1		2016.09.28 16:34:30	2016.09.28 07:34:30
		3.0		2016.10.02 20:53:07	2016.10.02 11:53:07
		3.3		2016.10.10 22:59:10	2016.10.10 13:59:10

Notes: UTC stands for Universal Time Coordinated. Earthquakes having magnitudes 2.0 or more occurred 157 times from September 12 to November 13, 2016.

1 The magnitudes were obtained from the Korea Meteorological Administration.

### Changes in Groundwater Level Caused by the M5.8 Earthquake

In addition to the geologic characterization made using rock cuttings, testing also involved geophysical logging with an acoustic televiewer (ATV) and an optical televiewer (OPTV) (Figure ^3^). Permeable zones occur from 18 to 22 m and from 73 to 76 m. Major fractures and joints are evident in the zone from 22 to 70 m, with minor joints occurring sporadically. The well has a surface casing to a depth of 15 m and is screened to the bottom of the hole at a depth of 100 m. A long‐term pumping test was carried out with the well to determine aquifer parameters.

**Figure 3:**
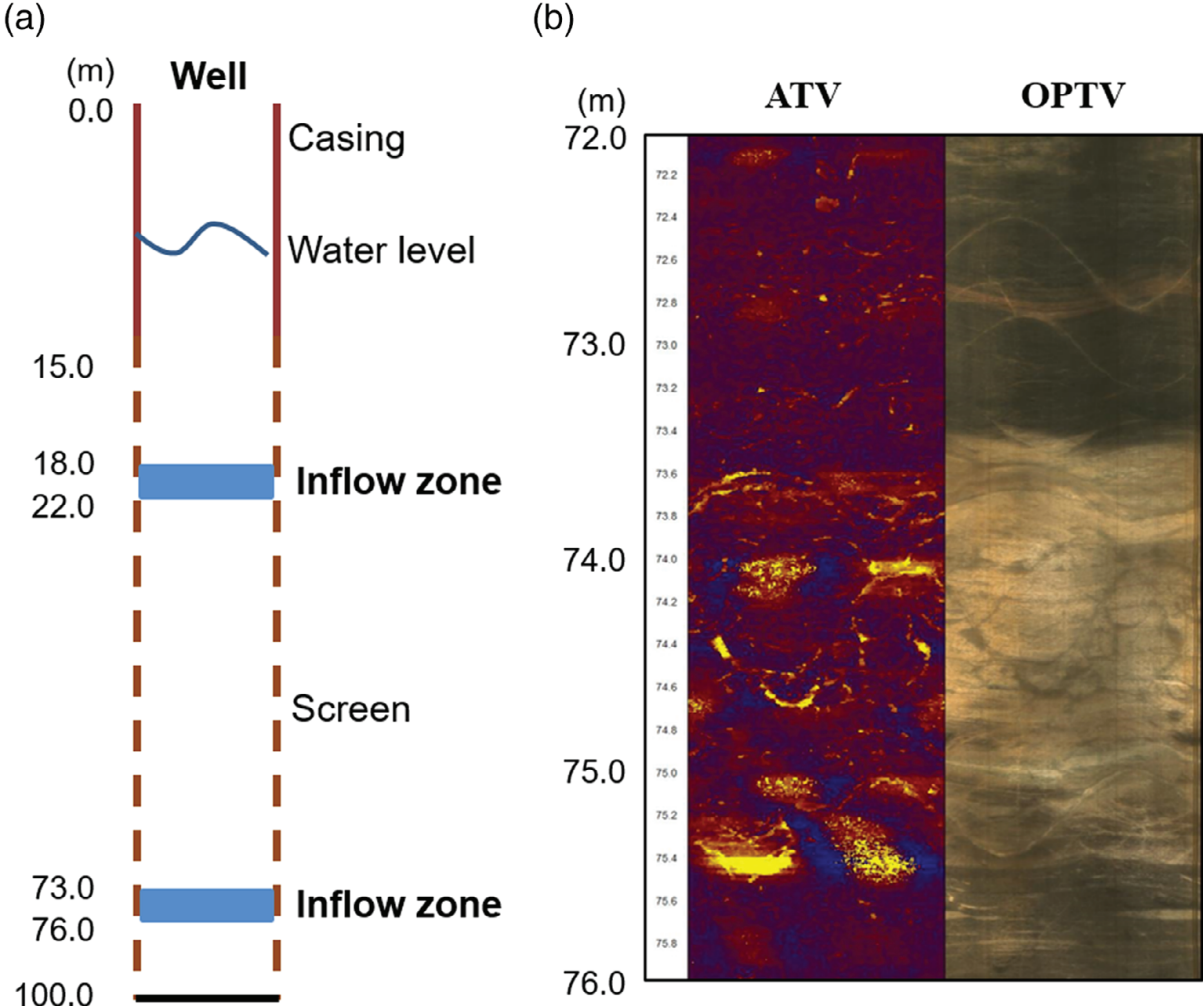
(a) A schematic diagram showing well construction details and zones of evident inflow to the well depicted as blue bars. (b) Fractures in the deeper inflow zone as determined by borehole logging with an ATV (left) and OPTV (right).

To interpret the test results, the Hantush solution for a wedge‐shaped confined aquifer was used to estimate hydraulic properties (Hantush 1962). This analytical solution assumes a wedge‐shaped confined aquifer that is infinite in extent with homogeneous and isotropic material properties. It also assumes that flow to the pumped well is horizontal, the thickness of the aquifer increases in the direction of flow, and that the rate of change in the aquifer thickness in the flow direction does not exceed 0.2 (= 11.3°). Calculations yielded a transmissivity estimate of 0.3334 m^2^/day.

Inspection of the data in Figure ^4^ indicates that the fluctuations in groundwater levels measured in MW 1 were normally associated with rain and tidal effects. However, the relatively large increase in water level around September 12 was significantly influenced by the M5.8(5.1) earthquakes and numerous aftershocks and to a lesser extent by the rain. There are three lines of evidence that support this supposition. First, the quantity of recorded rainfall was relatively small, 2.0, 14.1, and 47.9 mm on September 12, 16, and 17, respectively. These amounts were much lower than those at the end of June which triggered the rise observed before the earthquake. Second, over a longer period of time, there was an approximate 1.0 m rise after rainfalls totaled ∼190 mm in June and July, whereas the 1.5 m rise after the earthquake coincided with 140 mm of rainfalls. Finally, a M3.5 event occurred during a low‐rainfall period where levels were markedly declining. That event reversed the decline with an evident rise in water level and appears to have contributed to an obvious slowing in the overall rate of decline following the event (i.e., lower slope) (Figure ^4^). The water level increase a result of M5.8(5.1) earthquake and aftershocks is considered to be caused by a transient increase in hydraulic conductivity in the shallow rock units above 18 to 22 m zone by fracture reactivation or remobilization of fines that facilitated downward flow from shallower units (Brodsky et al. 2003; Claesson et al. 2007; Wang et al. 2009). The results of the pumping tests performed before and after the M5.8 Earthquake showed the increase of the transmissivity after the earthquake (Table 2).

**Figure 4:**
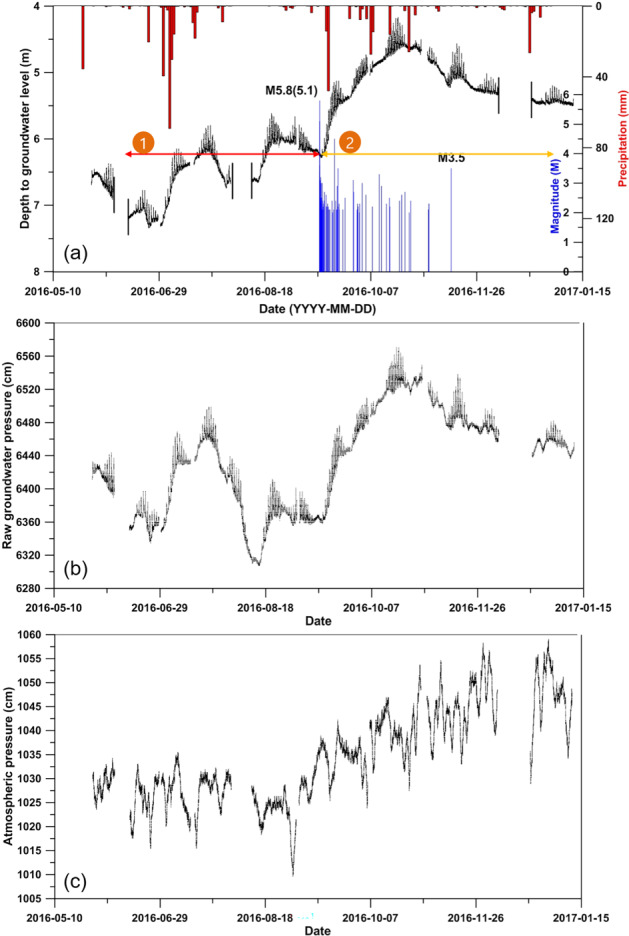
(a) Fluctuations in water level in the monitoring well over time (black lines). The small spikes represent tidal fluctuations. The blue histogram shows the timing and magnitude of aftershocks associated with M5.1, M5.8, and M3.5 earthquakes at the monitoring well from May 28, 2016 to January 10, 2017. The red histograms show precipitation in relation to the changing water levels and earthquake events. Fluctuations related with raw groundwater pressure (b) and atmospheric pressure (c).

**Table 2:** Results of Pumping Tests Before and After the M5.8 Earthquake

M5.8 Earthquake	Pumping Rate (m^3^/day)	Elapsed Time (min)	Drawdown (m)	Transmissivity (m^2^/day)
Before	50.0	496	77.1	0.3334
	27.5	1440	34.7	0.5629
After	25.0	183	7.1	0.6675
	38.0	900	29.5	0.4461

The method of spectral analysis (Rahi and Halihan 2013) was used to differentiate between groundwater level changes before the earthquake (June 1, 2016 to September 11, 2016) and those after the earthquake (September 16, 2016 to January 10, 2017).

Time series data, *x*_1_, …, *x*_*n*_, of size *n*, can be represented as follows (Shumway and Stoffer 2010):

$$x_{t}=a_{0}+\sum_{j=1}^{\left(n-1\right)/2}\left[a_{j}\cos\left(\frac{2\pi \mathrm{tj}}{n}\right)+b_{j}\sin\left(\frac{2\pi \mathrm{tj}}{n}\right)\right]$$

where *x* = time series data for the groundwater level measured at 1‐day interval, *t* = time, *n* = size of the time series data, and *j*/*n* = period of the *j*th harmonic component.

When *j*/*n* is not 0, 1/2, the regression coefficients (*a*_*j*_ and *b*_*j*_) in the above equation can be written as ($a_{0}=\bar{x})$:

$$a_{j}=\frac{2}{n}\sum_{t=1}^{n}x_{t}\cos\left(\frac{2\pi \mathrm{tj}}{n}\right)$$

$$b_{j}=\frac{2}{n}\sum_{t=1}^{n}x_{t}\sin\left(\frac{2\pi \mathrm{tj}}{n}\right)$$

Then the scaled periodogram ($P\left(\frac{j}{n}\right)$), measuring the presence of a frequency of oscillation of *j* cycles in *n*, is defined as follow:

$$P\left(\frac{j}{n}\right)=a_{j}^{2}+b_{j}^{2}$$

The spectral analysis shows significantly different cyclical components before the earthquakes as compared to 40 days later. In particular, after the earthquake events, a new long‐term period component was evident. It was longer than the existing periodic component (Figure ^5^a). A detailed analysis of daily changes in groundwater levels was carried out by detrending the data from May 28, 2016 to January 10, 2017. The results indicated a marked increase in groundwater level after the earthquake. Accounting also for differences in total amount of daily precipitation before and after earthquakes, it is believed that the increase in groundwater levels was mainly caused by the earthquake event (Figure ^5^b).

**Figure 5:**
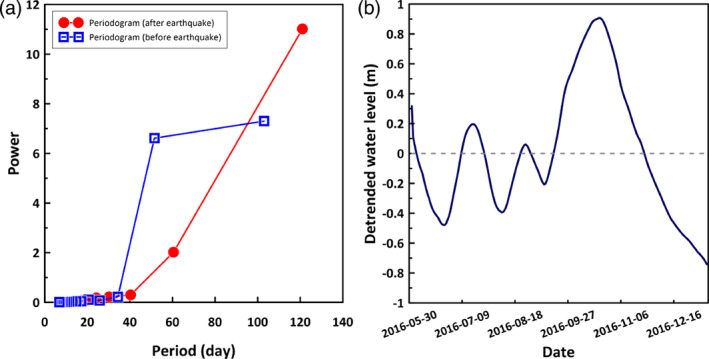
(a) Periodograms of groundwater level time series for the before earthquake (gray square: June 01, 2016 to September 11, 2016) and the after earthquake (black circle: September 12, 2016 to January 10, 2017). (b) Detrended fluctuation in groundwater levels from May 28, 2016 to January 10, 2017. Before the earthquake the daily events, intensity, and a total amount of rainfall are 26 days, 8.95 mm/day, and 232.7 mm, respectively, and after the earthquake 28 days, 7.96 mm/day, and 222.8 mm.

Figure ^6^ shows fluctuations in groundwater levels based on the observed data with the missing data estimated by a moving average. The reconstructed hydrograph is visually almost identical to the actual data displayed in Figure ^4^. The patterns of fluctuation in this reconstructed time series of groundwater levels were also analyzed by linear regression and neural networks. For the period from May 30 up to September 11, 2016 (before the earthquake), the groundwater levels exhibit a cyclical pattern of fluctuations within a distinct lower and upper bound 95% (red color). Following the earthquake, the pattern changed dramatically along with significant increase in water level (blue color). This change is interpreted as a transient inflow of groundwaters after the earthquake.

**Figure 6:**
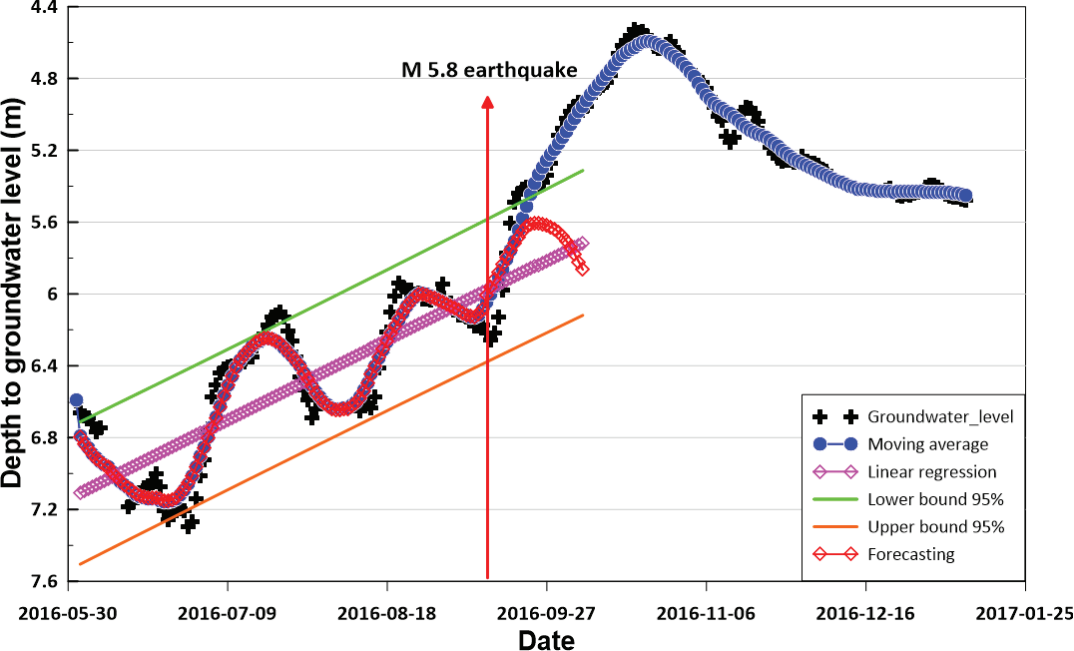
Forecasting time series using neural networks and linear regression to analyze association with earthquake events. Groundwater level significantly increases after the main earthquake (blue color) as comparing to levels before the event (red color).

### Vertical Profiles for Temperature and EC in the Monitoring Well

Vertical profiling of the water column with respect to temperature and EC showed that the monitoring well exhibited three different zones: (1) a freshwater or brackish water zone from the surface to 20 m; (2) a brackish water zone with EC between freshwater and deep saltwater from 20 to 80 m; and (3) a saline water zone below 80 m (Figure ^7^). Following the M5.8(5.1) earthquake, EC decreased in the brackish zone from an average of approximately 5300 to 4800 μS/cm, which can be caused by fracture reactivation or remobilization of fines that facilitated downward flow from shallower units above 18 to 22 m. Flow within the well displaced water upward and mixed with the casing water. With time, slower upward diffusion of water pushed the EC toward original values. The inflow cooler less brackish water to the well was also reflected in the temperature profile. Before the M5.8(5.1) earthquake there was a stable, steadily increasing temperature regime below 50 m (boundary point) reflecting geothermal effects (Figure ^8^). However, following the earthquake, the temperature of water below the boundary point was evidently cooler with temperatures declining slightly with depth. This behavior suggested the cooler inflow to the well through the fracture paths and then mixed. The vertical temperature profile in October after the earthquake occurred has returned to a pre‐earthquake regime, suggesting that water fluxes into the well were short lived and temperatures readjusted back to a geothermal gradient.

**Figure 7:**
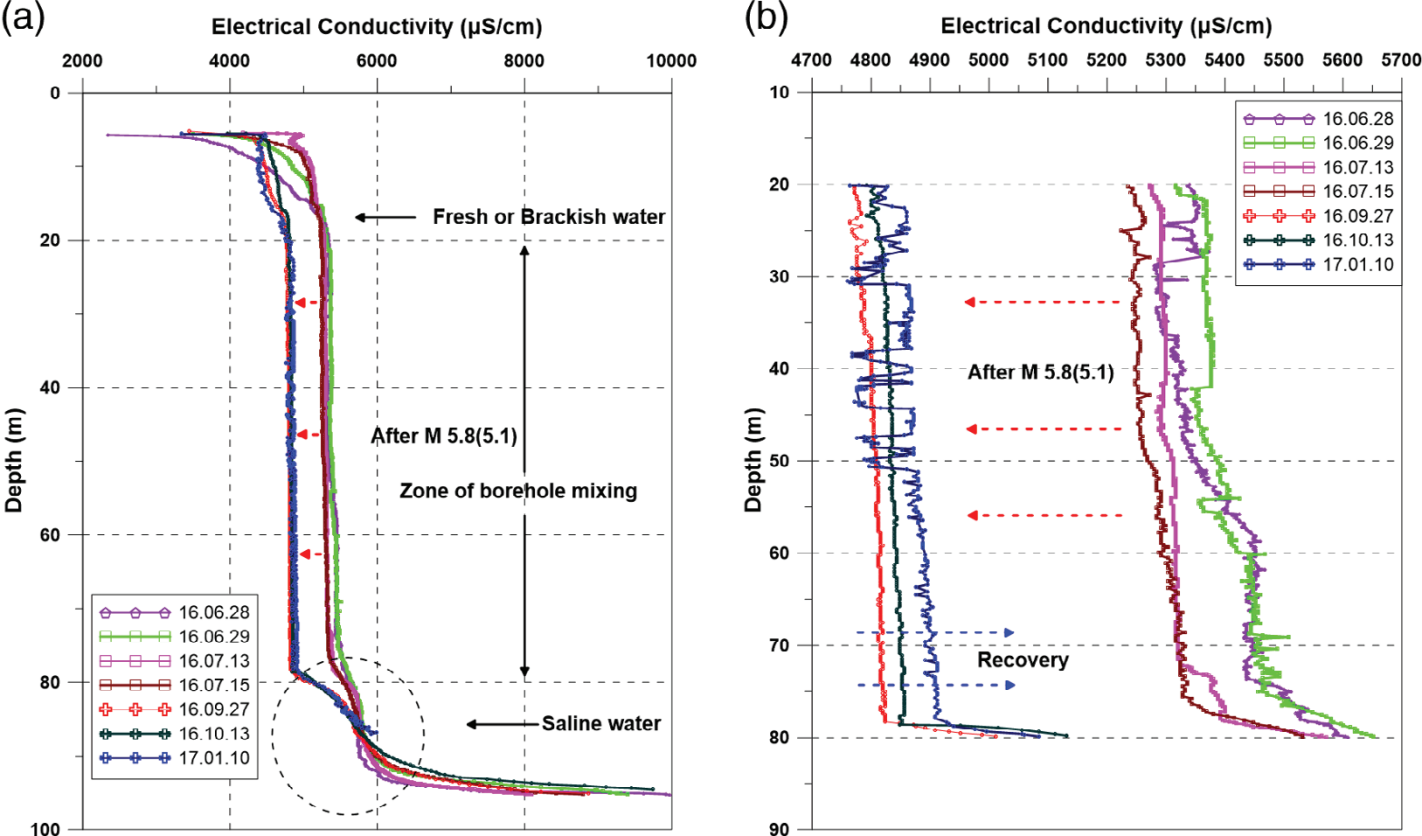
(a) Continuous vertical profiles in electrical conductivity for various depths (0 to 100 m). (b) A higher resolution version of the same profiles through the zone of borehole mixing. Notice both the tendency toward dilution following the main M5.8(5.1) Gyeongju earthquake and an indication of a recovery back to more saline conditions.

**Figure 8:**
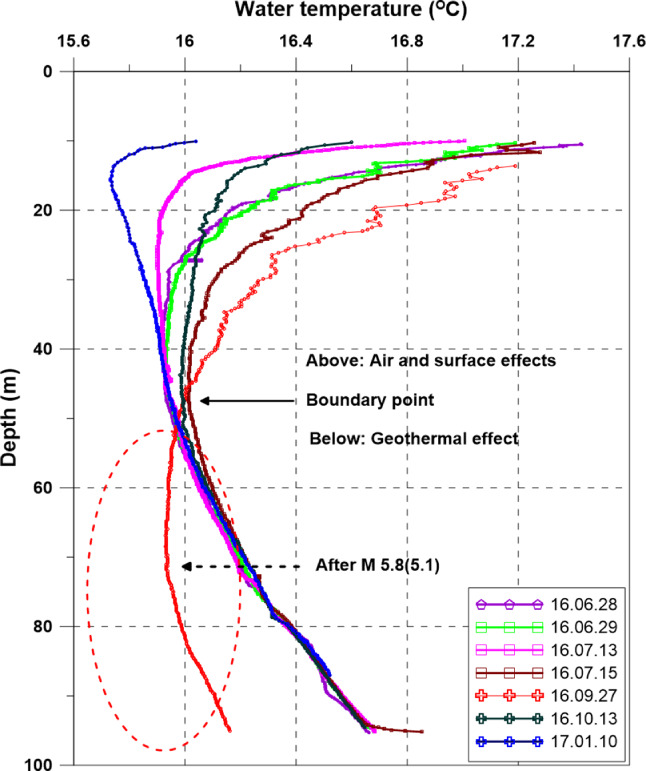
Vertical temperature profiles in the monitoring well for various dates before and after the M5.8(5.1) Gyeongju earthquakes.

The InterfacEGG device that is installed below 95 m detected a temporal change in the FSL, immediately after the M5.1 earthquake. The instrument was set up with a 5 min recording interval. An abrupt rise in EC was recorded 6 min after the first earthquake and the EGG rose continuously (Figure ^9^). This behavior reflects that the rise in EC where the EGG was positioned, was not due to the saltwater intrusion there, but from the tendency for brackish water entering the borehole to cause convective mixing at deeper depths as the seismic waves travel through the well‐aquifer system (Shi et al. 2007).

**Figure 9:**
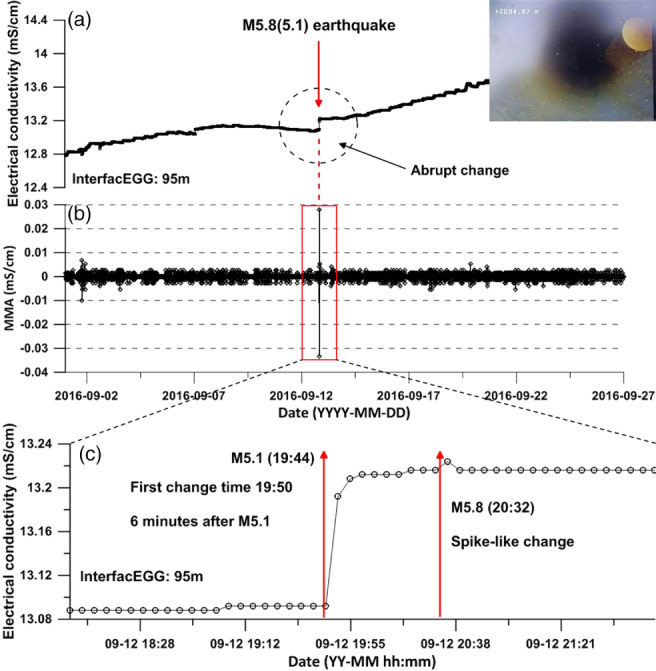
(a) Changes in electrical conductivity at the freshwater‐saltwater interface level located at 95 m in the months before and after the earthquake. (b) Filtered water level hydrograph that shows data on groundwater levels processed to remove tidal fluctuations and to correct for changing atmospheric pressures. (c) A highly resolved sequence of EC measurements with time, illustrating quick changes of the values following the earthquake.

The groundwater data (pressure, temperature, EC, etc.) contained various signals related to changes in atmospheric pressure, ocean tide, and earth tide. Modified moving average (MMA) filtering was performed to discriminate the responses to the earthquake effect at a certain time by removing the long‐term trend. Although the MMA filtering cannot remove the noise more sensitively than other methods, it can effectively and easily detect the instantaneous fluctuations caused by earthquakes. The following equation is used for these corrections:

$$X_{t}^{*}=X_{t}-\frac{X_{t-n}+\cdots X_{t}+\cdots X_{t+n}}{2n+1}$$

where $X_{t}^{*}$ is the filtered groundwater level, *X*_*t*_ is the raw groundwater level, and *n* is the time step; *n* = 1 was applied in this study. In previous studies, seismically induced groundwater fluctuations have been analyzed using the equation as a filtering method (Lee et al. 2013, 2017).

The observed changes in water level, the vertical profiles in temperature and EC, and the rise in EC measured with the InterfacEGG were mainly caused by the Gyeongju earthquake and aftershocks. The increase in groundwater levels was caused by pulse of colder, less brackish water flowing into the well because of the earthquake. This behavior reflects an enhancement in rock permeability by removing precipitates and colloidal particles from clogged fractures, which improve the hydraulic connection with a nearby unit with pore pressure changes. The in‐well mixing of this cooler less, water with the ambient water in the casing led to an overall decrease of EC and a temperature adjustment away from the normal geothermal gradient (Figure ^10^).

**Figure 10:**
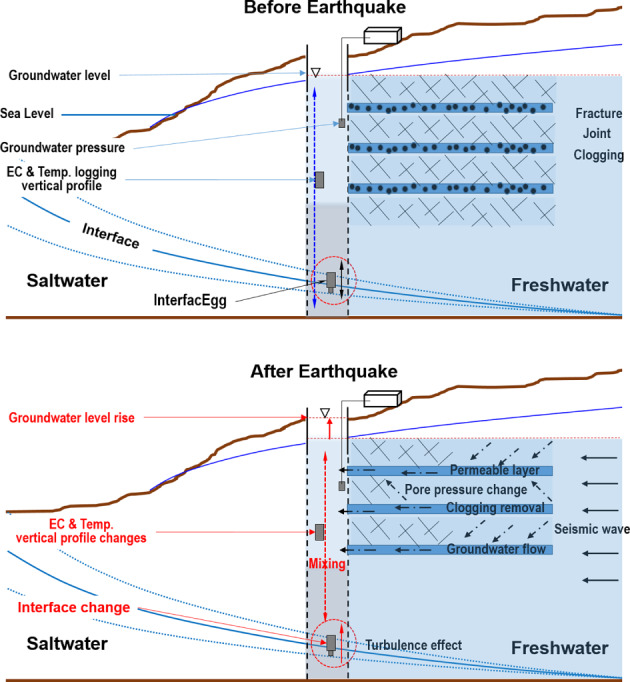
Conceptual model of changes in groundwater level and vertical profile in temperature and EC caused by the Gyeongju earthquake and aftershocks.

## Conclusions

Measurements from a deep observation well tapping a coastal aquifer revealed significant changes in groundwater levels and vertical profiles in temperature and EC (EC) caused by M5.8(5.1) Gyeongju earthquakes. Data analyses indicated that water flowed into the well through fractured zones. Changes in the physical and chemical characteristics of the water in the well were the result of a water pulse that caused an upward displacement of groundwater levels, and thus in the casing water. The nature of this water pulse could have been turbulent in nature. The array of instrumentation clearly detected the water pulses generated by the earthquake and aftershocks. Additional work will be required to elucidate details of this phenomenon.

This study established the benefits of using continuous well logging over single‐depth measurements of water level changes. The InterfacEGG proved to be useful in monitoring the freshwater‐saltwater interface and assessing the impact of the main earthquake on groundwater levels. Although our application was specifically concerned with water level changes associated with the impact of an earthquake, the potential usefulness of this monitoring system near coastal aquifers has been demonstrated.

## Authors' Note

The authors do not have any conflicts of interest.
